# Leveraging Translational Science to Disseminate Evidence on Using Herbs and Spices to Improve Diet Quality

**DOI:** 10.1093/nutrit/nuaf242

**Published:** 2026-05-26

**Authors:** Gail C D’Souza-Rushton, Olivia Lawler, Penny M Kris-Etherton, Kristina S Petersen, Travis D Masterson

**Affiliations:** Department of Nutritional Sciences, Pennsylvania State University, University Park, PA 16802, United States; Department of Nutritional Sciences, Pennsylvania State University, University Park, PA 16802, United States; Department of Nutritional Sciences, Pennsylvania State University, University Park, PA 16802, United States; Department of Nutritional Sciences, Pennsylvania State University, University Park, PA 16802, United States; Department of Nutritional Sciences, Pennsylvania State University, University Park, PA 16802, United States

**Keywords:** translational science, nutrition education, cooking, herbs and spices

## Abstract

Translational science bridges the gap between research and practice to positively influence health behaviors. Guided by core translational science principles—including producing solutions for a common challenge, emphasizing creativity, leveraging team science, and utilizing partnerships—our interdisciplinary team of educators, researchers, and clinicians at Pennsylvania State University (Penn State), with support from the McCormick Science Institute (MSI), has applied translational science approaches to address poor diet quality. Our recent activities have focused on translating previous research on herbs and spices alongside recommendations from the 2020-2025 Dietary Guidelines for Americans (DGA) to educate the general public about the benefits of herbs and spices to improve diet quality. Specifically, we developed evidence-based educational materials to teach the American public about using herbs and spices in home cooking. We also developed short and longer educational videos to assess the effectiveness of nutrition education videos provided to the American public, as well as to patients and medical professionals in a clinical setting. Across diverse audiences, results were consistent whereby the interest, knowledge, confidence, and intent to use herbs and spices in cooking increased in participants after watching the videos compared to before watching them. Participants also found the videos to be engaging and of high quality. These findings demonstrate the positive effects of short nutrition education videos and suggest that content can be condensed without losing effectiveness of impact. Building on this work, we hope to expand the implementation of evidence-based research into schools while also exploring the influences of messaging on eating behaviors among adolescents and their engagement with nutrition information through social media outlets. With support from MSI, we look forward to continuing this interdisciplinary collaboration and emphasizing creativity and innovation by utilizing cross-sectoral partnerships to produce new solutions to improve diet quality across all populations.

## INTRODUCTION

Translational science is the process of transforming laboratory research and observations from clinics and communities into practical interventions and messaging that can improve the health behaviors of individuals.^1^^,^^2^ A major goal of translational science is to enhance understanding of how to increase the speed at which research can be implemented in practice to improve public health.^2^ The National Center for Advancing Translational Sciences (NCATS) created the NCATS Translational Science Principles to be applied to research along the translational continuum.^1^ The NCATS Translational Science Principles consist of the following: (1) prioritizing initiatives that address unmet needs, (2) producing generalizable solutions for common and persistent challenges, (3) emphasizing creativity and innovation, (4) leveraging cross-disciplinary team science, (5) enhancing the efficiency and speed of translational research, (6) utilizing boundary-crossing partnerships, and (7) using bold and rigorous research approaches.^3^ Each of these principles helps researchers apply scientific and operational approaches to advance translation of information that is dependent on their field of research.^4^

Using translational science principles such as producing generalizable solutions for a common challenge, emphasizing creativity and innovation, leveraging cross-disciplinary team science, and utilizing boundary-crossing partnerships, our team of educators, researchers, and clinicians at Pennsylvania State University (Penn State), with support from the MSI, have been utilizing translational science approaches to address poor diet quality.

For example, obesity is a serious chronic disease that affects more than 2 in 5 adults in the United States.^5^ In 2023, obesity prevalence was recorded to be higher than 20% in all states of the United States and territories.^6^ Adults living with obesity are susceptible to other comorbidities such as high blood pressure, heart disease, and cancer that can lead to health complications and increased mortality.^6^ Obesity and its related comorbidities are highly correlated with diet quality, intake, and related nutrition behaviors.^7^ Specifically, the 2020-2025 Dietary Guidelines for Americans (DGA) reported that the average sodium intake in the United States ranges from 2000 to 5000 mg per day. Similarly, added sugars contributed to more than 13% of total calories per day for individuals in the United States, while intake of saturated fatty acids (SFAs) constituted 11% of calories. However, diet quality and lifestyle changes can be challenging to adopt and maintain. By use of a translational science lens, rather than continuing to generate data on the impact of diet on chronic disease outcomes, the goals to be addressed are to bridge the gap between research and practice and aid in creating robust practical applications of evidence-based research to enhance the sustainability of healthy dietary behaviors.^8^

Recent activities by our team have focused on translating previous research demonstrating that using herbs and spices to flavor foods lower in overconsumed dietary components such as sodium, added sugars, and SFAs preserves food acceptability and liking and may reduce overconsumption of these dietary components.^9–11^ Studies involving high school students have reported herbs and spices being likely to increase the consumption of nonprocessed foods like vegetables.^12^ Additionally, previous research has also highlighted the increased appreciation and intake of low-salt legume-based meals, which suggests that the addition of herbs and spices is a feasible option for achieving low salt content while maintaining liking of legumes among young adults.^13^ This research aligns with recommendations from the DGA to use herbs and spices to flavor foods lower in added sugars, sodium, and SFAs. Our research focused on developing evidence-based educational materials that address the use of herbs and spices in home cooking for the American public. These educational materials were tested on a broad, nationally representative sample, as well as in participants at medical clinics, to assess the impact and acceptance of these materials.^14^^,^^15^ In addition, we sought to understand whether these materials could be of use to clinicians in medical practice. In the following sections we expand on how these materials were developed by our cross-disciplinary team and how we considered translational principles throughout the development and research process. Additionally, we have highlighted potential future directions to be pursued in the advancement of improved diet, eating behaviors, and health.

## USING TRANSLATIONAL SCIENCE TO DEVELOP EFFECTIVE NUTRITION EDUCATION MATERIALS—NUTRITION EDUCATION VIDEOS

The 2020-2025 DGA highlighted the use of herbs and spices as a way of flavoring food to reduce the intake of sodium, added sugars, and SFAs. This guidance served as the basis for the design of our educational materials for several reasons: (1) the message is simple, (2) it allows substitutions rather than restrictions, (3) herbs and spices can be incorporated into many foods and cuisines. To develop these materials, we collaborated with faculty, graduate students, and Penn State Extension educators to develop a 1-hour webinar hosted by Penn State Extension titled, “Let’s Cook at Home: Herbs and Spices.” Leveraging cross-disciplinary team science with experts such as researchers, registered dietitians, and video producers helped us create an innovative webinar. The goal of this webinar was to help individuals reduce sodium, added sugars, and SFAs in their cooking by replacing them with herbs and spices. The webinar included live cooking demonstrations of 5 recipes to show that healthy cooking techniques can be easy.^14^ Each recipe was developed in collaboration with chefs from MSI to be delicious and beginner friendly, and to incorporate easily accessible herbs and spices. Some examples of the herbs and spices used included garlic powder, black pepper, and cumin. Additionally, the recipes were designed to be acceptable to a predominately White audience in Pennsylvania, as these individuals were more likely to attend the webinar hosted by our Extension team. The recipes included different parts of a meal (protein, grain, vegetarian, and dessert) along with an everyday seasoning that could be used on a wide variety of foods. Scripts, blocking, and website materials were designed by the interdisciplinary team for the webinar. The webinar also included a variety of Extension fact sheets and a question and answer session to involve participants.^14^

The webinar was marketed 1 month prior to launching and was viewed by 425 individuals who watched either the live or recorded webinar. After watching the webinar, participants were asked to complete a short questionnaire to assess their knowledge, confidence, and intention of using herbs and spices prior to and after watching the webinar.^14^ Overall, individuals had significant increases in their scores in knowledge (88%), confidence (88%), and interest (98%) variables. Additionally, participants were asked if they would be interested in getting more information and if short educational videos on these recipes would be useful. The majority (∼96%) of respondents reported that short (3-to-5-minute) videos detailing specific topics covered in the webinar would be useful for continued learning.^14^

With the identification of interest in short videos, the second step of this project was to develop educational videos and identify what content from the webinar would be best to feature.^14^ Extension educators worked alongside the research team to create scripts for 5 videos featuring information on the using herbs and spices to flavor recipes lower in sodium, added sugars, and SFAs.^14^ Additional content included was how to read nutrition facts labels, recommended intake for salt, how to make spice blends at home, and safe food handling.

After development, the videos were evaluated by the general public using an online sample. Similar to the webinar study, this study sought to assess whether the nutrition education videos could increase knowledge, confidence, and intention to use herbs and spices in home cooking. Overall, the video materials were successful, with participants reporting significant increases in knowledge, confidence, and intention to use herbs and spices to reduce sodium, added sugars, and SFAs.^14^ As the educational materials showed promise with the general population there was a need to make them available to ensure that the translational impact of the videos did not stop at the development stage. Therefore, the videos were posted on the USDA Partner Resources website (https://www.myplate.gov/partner-resources) to be made widely available to the public. The videos are also available on the Penn State Extension website (Resource Page for Let’s Cook at Home: Herbs and Spices; https://extension.psu.edu/lets-cook-with-herbs-and-spices). Additionally, Penn State Extension continued to use these videos and accompanying fact sheets from the webinar in their community outreach programs.

## EXTENDING TRANSLATIONAL SCIENCE TO a MEDICAL SETTING

Using the videos created from the previous project, we sought additional dissemination routes to increase the translational reach of our educational materials. We identified the medical office setting as a potential channel for dissemination and sought to test the effectiveness of nutrition education videos compared to traditional handout materials as a means to educate patients about the use of herbs and spices during cooking. In addition, we also surveyed medical students and medical professionals in family and community health settings to ascertain their opinions of the materials. These studies allowed us to better understand how educational materials could be distributed in clinics and the potential impact of these materials on interest, knowledge, confidence, and the intent of the students to use herbs and spices in a patient population.^15^ For these studies, patients and providers (medical students and health care providers) from Penn State Health family medicine outpatient clinics participated. Utilizing cross-sectoral partnerships with existing providers at Penn State Health allowed us to conduct our study seamlessly. For the patient study, patients were randomized to view either 5 short videos or read 3 handouts.^15^ A survey was conducted before participants were randomized to the study while they were waiting to see their provider. Once the patients viewed the videos or read the handouts, they were provided with another survey that was then completed on the patients’ smartphone at their convenience. The majority of participants racially identified as White (86%) and were Non-Hispanic or Latino (84%). Results from this study showed higher scores from the video group than the handout group for interest, knowledge, confidence, and intent to use herbs and spices.^15^ Patients also reported that the videos were clearer when compared to the handout materials even though the materials were similar and created by the same education team.^15^ However, some patients did not have time to view all of the videos within the time they had been waiting for their provider. This situation demonstrated the need for our team to develop shorter videos that could still retain the educational content we intended to disseminate. Nonetheless, results from this study demonstrated the potential benefits of multimedia approaches to nutrition education in health clinics and preventive healthcare.

From the unpublished providers study data, we found that both medical students and providers had increased interest, knowledge, confidence, and intent to use herbs and spices when preparing a meal after viewing the educational videos compared to before. Additionally, on average the participants rated the videos as high quality and providers indicated that the videos would meet the needs of their patients. Medical providers also saw the value of using the educational videos in their clinics during patient wait times. Overall, the videos were an effective educational tool for use in clinics and were well accepted by medical providers.

## CONTINUED TRANSLATION AND TESTING OF NUTRITION EDUCATION VIDEOS

Following the results from the studies in the medical clinics we noted that although the materials were well accepted and appeared to be effective, the videos needed to be shortened to meet the needs of wait times in clinics. Additionally, in the public sphere, popular social media outlets such as YouTube and TikTok are increasingly moving toward short form media (ie, < 1 minute of content). Therefore, for both clinical and general dissemination our team sought to understand if short (∼1 minute) nutrition education videos could be as effective as longer (∼5 minute) videos.^16^

The process of creating short videos encouraged us to leverage creativity and innovation in our research practices. Both the longer and short videos included similar content regarding recipe information and instruction, but the duration differed.^16^ The videos featured recipes lower in sodium, added sugars, and SFAs with herbs and spices used as replacements. Similar to our previous work, we saw that regardless of video length, participant interest, knowledge, confidence, and intent to use herbs and spices in their cooking increased after watching the videos compared to before watching them.^16^ The survey also inquired about participant perceptions of the videos and their barriers to using herbs and spices in their cooking. Participants in both conditions also reported that both videos were interesting and engaging and conveyed educational information that was easy to follow and understand, and that the videos were an effective method to increase the use of herbs and spices in cooking.^16^ Although we hypothesized that shorter videos would be better received by participants, our analysis revealed no statistically significant differences between short and longer videos. This finding demonstrated that the content of the videos could be successfully condensed without losing effectiveness.^16^

## FUTURE DIRECTIONS

The majority of our research has focused on using nutrition education videos to provide information to the general population on how to use herbs and spices as a replacement for sodium, added sugars, and SFAs in cooking at home. In the future, we would also like to understand how messaging can influence the use of herbs and spices in cooking. Our studies have focused on messaging related to the health benefits of reducing sodium, added sugars, and SFAs, but other forms of messaging may also resonate with viewers. For example, taste-based messaging may appeal more to some audiences, particularly those who are less health conscious. It is well established that taste is a leading driver of food choice. Further, the perception of the poor taste of healthier foods, which has been described as the “*unhealthy = tasty intuition*,” is well documented and posits that the less healthy an item is portrayed to be, the better the inferred taste and the more it is enjoyed.^17^ Therefore, assessing the effectiveness of taste vs health messaging will advance knowledge of content messaging strategies to motivate dietary behavior change. Another approach would be to survey parents and adolescents about their sources of nutritional education, assess adolescent acceptance of recipes and preferred messaging outlets to encourage individuals of this age group to eat healthier foods. This information can help us gain insight on the impact of social media messaging on adolescents and eventually utilize social media to engage with adolescents about the benefits of healthy eating behaviors.

Building on our prior research demonstrating that the addition of herbs and spices to enhance the flavor of commonly consumed recipes reformulated to be lower in sodium, added sugars, and SFAs is acceptable to consumers and has the potential to lower intake of these overconsumed dietary components, we are now aiming to translate these findings to the school setting.^9^ This is based on a need identified because of the 2024 release of the Final Rule on Child Nutrition Programs.^18^ This rule introduced limits to added sugar and updates to total sugar and sodium limits. During the public comment period, stakeholders, including school nutrition professionals, expressed concerns about the time and resources needed for reformulation to meet the new standards as well as the acceptance by students of foods lower in sugars and sodium. Therefore, we aim to acceptably reformulate commonly used recipes in schools to meet the new sugar and sodium targets and facilitate implementation by school nutrition programs across the United States. We also aim to conduct feasibility studies to increase understanding of barriers and facilitators to implementing these reformulated recipes using the Consolidated Framework for Implementation Research. Our goal is to interview and survey school administrators and school staff to inquire about the barriers and facilitators to implementing the new recipes.

Using translational science principles has allowed us to develop evidence-based educational tools for use in different settings, such as medical clinics and schools. The collaborative translational science efforts between Penn State and MSI have benefited the research community by promoting generalizable solutions such as nutrition education videos to educate Americans about using herbs and spices in cooking. This research exemplified cross-disciplinary team science by integrating expertise from the Penn State Department of Nutritional Sciences; Penn State Extension; WPSU; and Health, Ingestive Behavior, and Technology laboratory to create and disseminate the videos to the public through the United States Department of Agriculture (USDA) website. Through boundary-crossing partnerships with industry collaborators like MSI, our team has demonstrated the potential benefits of using translational science to disseminate evidence about using herbs and spices to improve diet quality. These partnerships can facilitate the translation of scientific evidence-based research into practice and also enhance our health communications to benefit public health.

## References

[1] NCATS. About Translational Science. Accessed April 25, 2025. https://ncats.nih.gov/about/about-translational-science

[2] Curry SH. Translational science: past, present, and future. Biotechniques. 2008;44:ii-viii. 18422489 10.2144/000112749

[3] Faupel‐Badger JM , Vogel AL, Austin CP, Rutter JL. Advancing translational science education. Clin Transl Sci. 2022;15:2555-2566. 36045637 10.1111/cts.13390 PMC9652430

[4] National Center for Advancing Translational Sciences (NCATS). Translational Science Principles. Accessed April 25, 2025. https://ncats.nih.gov/about/about-translational-science/principles

[5] Centers for Disease Control and Prevention. Adult Obesity Facts. Accessed November 20, 2025. https://www.cdc.gov/obesity/adult-obesity-facts/index.html

[6] Centers for Disease Control and Prevention. Adult Obesity Prevalence Maps. Updated September 21, 2023. https://www.cdc.gov/obesity/data-and-statistics/adult-obesity-prevalence-maps.html?CDC_AAref_Val=https://www.cdc.gov/obesity/data/prevalence-maps.html

[7] Artinian NT , Fletcher GF, Mozaffarian D, et al; American Heart Association Prevention Committee of the Council on Cardiovascular Nursing. Interventions to promote physical activity and dietary lifestyle changes for cardiovascular risk factor reduction in adults: a scientific statement from the American Heart Association. Circulation. 2010;122:406-441. 20625115 10.1161/CIR.0b013e3181e8edf1 PMC6893884

[8] Ioachimescu OC , Shaker R. Translational science and related disciplines. J Investig Med. 2025;73:3-26. 10.1177/10815589241283515 PMC12097275 39262144

[9] Petersen KS , Fulgoni VL, Hopfer H, Hayes JE, Gooding R, Kris-Etherton P. Using herbs/spices to enhance the flavor of commonly consumed foods reformulated to be lower in overconsumed dietary components is an acceptable strategy and has the potential to lower intake of saturated fat and sodium: a national health and nutrition examination survey analysis and blind tasting. J Acad Nutr Diet. 2024;124:15-27.e1. 10.1016/j.jand.2023.07.025 37532099

[10] Peters JC , Marker R, Pan Z, Breen JA, Hill JO. The influence of adding spices to reduced sugar foods on overall liking. J Food Sci. 2018;83:814-821. 10.1111/1750-3841.14069 29476623 PMC5873279

[11] Polsky S , Beck J, Stark RA, Pan Z, Hill JO, Peters JC. The influence of herbs, spices, and regular sausage and chicken consumption on liking of reduced fat breakfast and lunch items. J Food Sci. 2014;79:S2117-26. 10.1111/1750-3841.12643 25219391 PMC4197100

[12] D’Adamo CR , Parker EA, McArdle PF, et al The addition of spices and herbs to vegetables in the National School Lunch Program increased vegetable intake at an urban, economically-underserved, and predominantly African-American high school. Food Qual Prefer. 2021;88:104076. 32999533 10.1016/j.foodqual.2020.104076 PMC7521666

[13] Dougkas A , Vannereux M, Giboreau A. The impact of herbs and spices on increasing the appreciation and intake of low-salt legume-based meals. Nutrients. 2019;11:2901. 10.3390/nu11122901 PMC6950109 31805675

[14] Lawler O , Masterson T, Petersen K, et al Online nutrition education videos teaching how to use herbs and spices to improve diet quality. J Nutr Educ Behav. 2024;56:672-677. 10.1016/j.jneb.2024.05.231 38934961

[15] Johnston A , Voulo M, D’Souza GC, et al Nutrition education in primary care: comparing video vs handout interventions. J Nutr Educ Behav. 2025;57:141-147. 10.1016/j.jneb.2024.09.008 39530959

[16] D’Souza-Rushton GC , Long JW, Denmon A, Kris-Etherton PM, Petersen KS, Masterson TD. Nutrition education “shorts”: the effect of short-form media on conveying information about improving diet quality. Nutrients. 2025;17:1612. 40431351 10.3390/nu17101612 PMC12114486

[17] Raghunathan R , Naylor RW, Hoyer WD. The unhealthy = tasty intuition and its effects on taste inferences, enjoyment, and choice of food products. J Mark. 2006;70:170-184.

[18] USDA. Final Rule—Child Nutrition Programs: Meal Patterns Consistent With the 2020-2025 DGAs. Accessed November 20, 2025. https://www.fns.usda.gov/cn/fr-042524

